# Importance of anemia in heart failure over blood pressure variability

**DOI:** 10.1002/clc.24141

**Published:** 2023-08-30

**Authors:** Shunsuke Kiuchi, Shinji Hisatake, Takayuki Kabuki, Shintaro Dobashi, Yoshiki Murakami, Takanori Ikeda

**Affiliations:** ^1^ Department of Cardiovascular Medicine Toho University Faculty of Medicine Tokyo Japan

**Keywords:** anemia, blood pressure in‐hospital variability, cardio ankle–brachial index, heart failure, prognosis

## Abstract

**Background:**

The evaluation of arteriosclerosis (vascular function) is important when treating heart failure (HF). Vascular dysfunction is associated with anemia through renal function and endothelial nitric oxide synthase. Additionally, blood pressure (BP) variability (BPV) caused by vascular dysfunction is also associated with HF prognosis. However, how anemia and BPV may affect HF prognosis is unclear.

**Methods:**

Between January 2012 and July 2018, 214 patients with HF were hospitalized. The cardio‐ankle vascular index (CAVI) as an index of arteriosclerosis of these patients was measured. The patients were divided into the elevated and preserved CAVI groups. We investigated the factors related to major adverse cardiovascular events (MACEs) as cardiovascular death or rehospitalization within 1 year after discharge.

**Results:**

In the elevated CAVI group, significant differences in body mass index (BMI), BPV, left ventricular dimension, and hemoglobin levels were observed between patients with and without MACEs. In the preserved CAVI group, significant differences in BMI, diastolic/mean BP, and hemoglobin levels were observed between those with and without MACEs. The multivariate analysis showed an independent association between hemoglobin levels and MACE occurrence in both the elevated and preserved CAVI groups (elevated CAVI group: hazard ratio [HR] = 0.800, *p* = .045 [model 1], HR = 0.802, *p* = .035 [model 2]; preserved CAVI group: HR = 0.783, *p* = .049 [model 1], HR = 0.752, *p* = .023 [model 2], and HR = 0.754, *p* = .024 [model 3]).

**Conclusions:**

Anemia was independently associated with HF prognosis with or without arteriosclerosis.

## Graphical abstract

In both the elevated (A) and preserved (B) cardio‐ankle vascular index (CAVI) groups, patients with low Hb levels had more major adverse cardiovascular events (MACEs) 1 year after discharge of acute decompensated heart failure (grouping according to the median Hb level). The multivariate analysis showed an independent association between hemoglobin levels and MACE occurrence in both the elevated and preserved CAVI groups (C; elevated CAVI group: hazard ratio [HR] = 0.800, *p* = .045 [model 1], HR = 0.802, *p* = .035 [model 2]: D; preserved CAVI group: HR = 0.783, *p* = .049 [model 1], HR = 0.752, *p* = .023 [model 2], and HR = 0.754, *p* = .024 [model 3]).

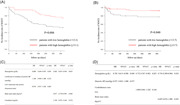

**Abbreviations**
ABI: ankle–brachial index
ADHF: acute decompensated heart failure
BMI: body mass index
BNP: brain natriuretic peptide
BP: blood pressure
BPV: blood pressure variability
CAVI: cardio‐ankle vascular index
CS: clinical scenario
CTR: cardiothoracic ratio
CV: coefficient of variation
eGFR: estimated glomerular filtration rate
ESAs: erythropoiesis‐stimulating agents
Hb: hemoglobin
HDL: high‐density lipoprotein cholesterol
HF: heart failure
HFpEF: heart failure with preserved ejection fraction
HFrEF: heart failure with reduced ejection fraction
ID: iron deficiency
LDL: low‐density lipoprotein cholesterol
MACE: major adverse cardiovascular events
NYHA: New York Heart Association classification
PP: pulse pressure
PWV: pulse wave velocity
RAS‐Is: renin–angiotensin–aldosterone system inhibitors
SD: standard deviation
TG: triglyceride
TTE: transthoracic echocardiography

## 1 INTRODUCTION

The heart failure (HF) pandemic is an issue that refers to a growing number of patients with HF. By 2030,^1^ the number of patients with HF in Japan is projected to reach approximately 1.3 million. The high readmission rates due to HF have been reported to have the capacity to increase in‐hospital and postdischarge mortality.^2^, ^3^ Reducing HF readmissions is necessary to improve prognosis. However, HF readmissions have not decreased despite advances in therapeutic methods.^4^ One reason is that vascular function is also involved in the pathogenesis of HF.^5^

Impaired vascular function is associated with anemia through renal function and endothelial nitric oxide synthase.^6^ Many patients with HF have anemia. Anemia in HF is related to severe symptoms and increased HF rehospitalization or mortality.^7^ These results are essential for all HF types, including HF with reduced ejection fraction (EF) (HFrEF) and HF with preserved EF (HFpEF). Moreover, blood vessels repeatedly contract and expand with cardiac contractions and moderate blood pressure (BP) fluctuations.^8^ Thus, when vascular function is impaired, the range of BP fluctuations expands. Additionally, BP variability (BPV) is associated with HF prognosis.^9^

However, how anemia and BPV affect HF prognosis is unclear. In this study, we evaluated how anemia and BPV affected the 1‐year prognosis of patients with HF classified according to their vascular function.

## 2 MATERIALS AND METHODS

This study was approved by the Ethics Committee of Toho University Omori Medical Center (approval number: M21292_M18271_17318). This study was a retrospective observational study conducted at a single center according to the Declaration of Helsinki (the medical records were accessed for data collection). The medical records of the patients were accessed between January 2020 and June 2022. After that, the collected data were analyzed for research. We have publicized the implementation of the clinical research on the electronic bulletin board in Toho University Omori Medical Center, and we have posted the details of the study in an opt‐out format on the website of Toho University Omori Medical Center and our department (Department of Cardiovascular Medicine). The subjects for this study were disclosed and provided the opportunity to decline to be further enrolled in the study (opt‐out). Because data were evaluated retrospectively and pseudonymously and were solely obtained for treatment purposes, the requirement of informed consent was waived by the Ethics Committee of the Toho University Omori Medical Center (opt‐out).

### 2.1 Study subjects

In this study, we used the cardio‐ankle vascular index (CAVI) to investigate vascular function. We recruited 214 patients with acute decompensated HF (ADHF) hospitalized at our institution between January 2012 and July 2018, who met the inclusion criteria for the study. We diagnosed ADHF using the guidelines of the American Heart Association or the European Society of Cardiology.^10^, ^11^ Subjects whose ankle–brachial index (ABI) was <0.9 or >1.4 were excluded. Furthermore, those who died at the hospital or were transferred to another hospital were excluded. A flowchart of the recruitment of the subjects is shown in Supporting Information: Figure.

### 2.2 Study outcomes

In this study, the main outcome was the relationship between major adverse cardiovascular events (MACEs) at 1 year, anemia, and BPV. MACE was defined as cardiovascular death or rehospitalization due to ADHF. The variables in patients with MACEs were compared with those in patients without MACEs and between the elevated and preserved CAVI groups. The elevated CAVI group consisted of patients with CAVI ≥ 9.0, and the preserved CAVI group comprised patients with CAVI < 9.0. Furthermore, multivariate analysis was performed to predict the occurrence of MACEs.

### 2.3 Clinical and physical profiles

We investigated age, sex, weight, height, New York Heart Association classification (NYHA), hospital stay, BP, and heart rate. We also calculated the body mass index (BMI) using the following formula: BMI = weight (kg)/height^2^ (m^2^). The patients were assessed for clinical scenario classifications (CS) based on systolic BP.^12^ On admission, the systolic and diastolic BPs was calculated using an aneroid sphygmomanometer. The mean BP and pulse pressure (PP) were calculated using the following formula: PP = systolic BP − diastolic BP and mean BP = diastolic BP + [PP/3]. BP during hospitalization was measured three times or more by a nurse (at 6:00, 10:00, and 18:00). In‐hospital BPV was evaluated using BP values (measured eight times or more) for 3 days before discharge when the condition was stable. We calculated the coefficient of variation (CV) and standard deviation (SD) of systolic BP to evaluate in‐hospital BPV.^13^ CV was defined as the within‐patient SD divided by BP values.^14^ Standard 12‐lead electrocardiography was performed to assess the heart rate in the supine position on admission. Moreover, the medical history of hypertension, diabetic mellitus, or atrial fibrillation or paroxysmal atrial fibrillation, chronic kidney disease, and hemodialysis was investigated. Hypertension was defined as the administration of antihypertensive medications or systolic BP ≥ 140 mmHg or diastolic BP ≥ 90 mmHg. Similarly, diabetic mellitus was defined as the administration of hypoglycemic medications or hemoglobin (Hb) A1C ≥ 6.5. Chronic kidney disease was defined as an estimated glomerular filtration rate (eGFR) <60 at least once before admission.

### 2.4 Physiological functional examinations

For physiological and functional examinations, transthoracic echocardiography (TTE) was performed and the CAVI was measured. Two physicians blinded for the study performed TTE. Left ventricular systolic function and left ventricular end‐systolic/end‐diastolic and left atrial dimensions were used to measure heart size. The EF was calculated using either the modified Simpson method (apical two‐ or four‐chamber view) or the Teichholz method (parasternal long‐axis view).^15^ Furthermore, the proportion of patients with HFpEF was also evaluated.^16^ HFpEF was defined as EF > 50% according to the guidelines from the Japan Circulation Society, the American Heart Association, and the European Society of Cardiology.^17^, ^18^, ^19^

VaSera VS‐1500E (Fukuda Denshi Company, Ltd.) was used to measure the ABI and CAVI after 12 h of fasting in the morning following ADHF improvement.^20^ We defined ADHF improvement as a general state that allows for hospital discharge. After 10 minute in the supine position, the patients' bilateral upper arms and ankles were cuffed, and their electrocardiogram, and heart sounds were monitored. The pulse wave velocity (PWV) can be calculated by dividing the length of the vascular system by the amount of time it takes for the pulse wave to travel from the aortic valve to the ankle. The CAVI was calculated as follows: CAVI = a((2ρ × 1/(sBP − dBP)) × (In(sBP/dBP) × PWV2)) + b; where ρ is blood density and a and b are constants to match aortic PWV. The left and right CAVI values were averaged to determine the CAVI.

### 2.5 Laboratory findings and chest *X*‐ray

We analyzed the laboratory results for C‐reactive protein, liver function (i.e., aspartate aminotransferase, alanine aminotransferase, and lactate dehydrogenase), Hb, electrolytes (i.e., sodium and potassium), brain natriuretic peptide (BNP), and renal function (i.e., ceGFR, blood urea nitrogen, and creatinine). The eGFR was calculated using the Japanese Society of Nephrology criteria, as follows: eGFR = 194 × Cr − 1.094 × age − 0.287 for men and 194 × Cr − 1.094 × age − 0.287 × 0.739 for women.^21^ Furthermore, the rate of anemia was evaluated. Anemia was defined according to the World Health Organization criteria as Hb < 13.0 g/dL in men and <12.0 g/dL in women.^22^ We evaluated triglyceride (TG), high‐density lipoprotein cholesterol (HDL) and low‐density lipoprotein cholesterol (LDL) as lipid metabolism markers. LDL was essentially measured directly. If LDL was not measured directly, LDL was calculated with the Friedewald formula: LDL = Total cholesterol − HDL − TG/5).^23^ We also evaluated fasting plasma glucose and Hb A1C as glucose metabolism markers. We assessed the chest *X*‐ray obtained on admission to determine the cardiothoracic ratio (CTR). Two blinded physicians calculated the CTR using the maximal cardiac and intrathoracic diameters.

### 2.6 Concomitant medications

The use of cardioprotective medications, such as β‐blockers, renin–angiotensin–aldosterone system inhibitors (RAS‐Is), and mineralocorticoid receptor antagonists, was evaluated. RAS‐I was defined as angiotensin‐converting‐enzyme inhibitors or angiotensin II type 1a receptor blockers. We investigated the rates of administration of cardioprotective medications at discharge. For β‐blockers and RAS‐Is, we investigated the proportion of patients who received ≥50% of each maximum dose from the package insert information.

### 2.7 Statistical analysis

Data are presented as medians or means ± SDs. We used the Mann–Whitney U test or the unpaired Student's *t*‐test to compare the groups. In all cases, differences with *p* < .05 were considered statistically significant. Excel (Microsoft XP) and EZR, a graphical user interface for R, version 2.13.0 (The R Foundation for Statistical Computing, Vienna, Austria), were used for all statistical analyses and were run on a Windows computer.^24^

## 3 RESULTS

### 3.1 Clinical and physical profiles in both groups

Supporting Information: Tables 1 and 2 show the clinical and physical profiles. No statistically significant differences in age and sex were observed between the groups; however, in both the elevated and preserved CAVI groups, the physique of patients with MACEs was significantly smaller than that of those without MACEs. In the elevated CAVI group, no significant difference in the BP values was observed. However, patients with MACEs showed significant changes in the CV, indicating BPV. In contrast, in the preserved CAVI group, the diastolic, and mean BPs of patients with MACEs were higher than those of patients without MACEs, although the SD, and CV showed no significant differences. Furthermore, no significant differences in medical history were observed.

### 3.2 Physiological examination and severity of HF

Supporting Information: Tables 3 and 4 show the examination results for patients with and without MACEs in the elevated and preserved CAVI groups. No difference in HF severity represented by BNP and NYHA was observed between patients with and without MACEs. Additionally, no significant difference in CS and the proportion of patients with HFpEF was observed between the two groups, indicating a similarity in the pathophysiology of HF. The left ventricle was enlarged in patients without MACEs; however, only the elevated CAVI group showed a significant difference. The ABI and CAVI of patients without MACEs were comparable to those of patients with MACEs.

### 3.3 Anemia and MACEs

**Figure 1:**
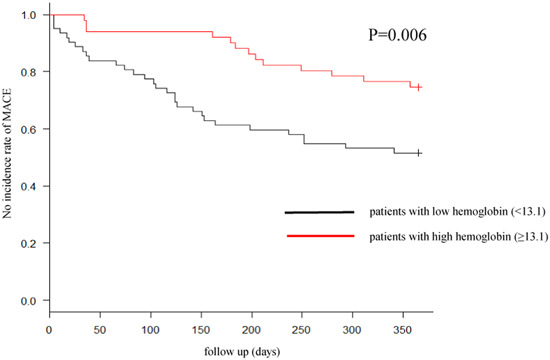
MACE within 1 year after discharge in the elevated CAVI group. MACE within 1 year after discharge in patients with low hemoglobin (<13.1 g/dl) was significantly higher than high hemoglobin (≥13.1 g/dl). *p* Values were determined using the log‐rank test. CAVI, cardio ankle vascular index; MACE, major adverse cardiovascular events.

**Figure 2:**
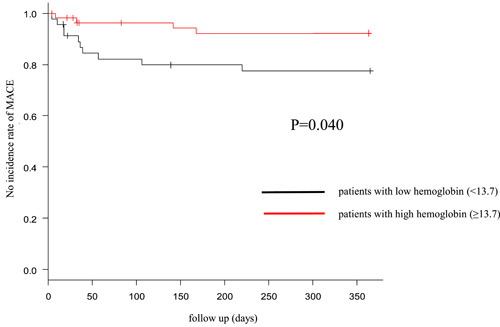
MACE within 1 year after discharge in the preserved CAVI group. MACE within 1 year after discharge in patients with low hemoglobin (<13.7 g/dl) was significantly higher than high hemoglobin (≥13.7 g/dl). *p* Values were determined using the log‐rank test. CAVI, cardio ankle vascular index; MACE, major adverse cardiovascular events.

In the elevated CAVI group, the Hb levels of patients with MACEs were significantly lower than those of patients without MACEs (MACE: 12.4 ± 2.4 g/dL vs. non‐MACE: 13.6 ± 1.8 g/dL; *p* = .003). Moreover, patients with MACE2 had a significantly higher proportion of those with anemia (MACE: 51.2% vs. non‐MACE: 28.6%; *p* = .016). These findings were similar in the preserved CAVI group (Hb: MACE: 12.4 g/dL vs. non‐MACE: 14.0 g/dL, *p* = .019; the proportion of those with anemia: MACE: 57.1% vs. non‐MACE: 22.9%, *p* = .041). Multivariate analysis showed that Hb was independently associated with the occurrence of MACE in both groups (Tables 1 and 2). In both the elevated and preserved CAVI groups, patients with low Hb levels had more MACEs 1 year after discharge (Figures 1 and 2; grouping according to the median Hb level).

**Table 1:** Multivariate analysis for prediction of MACE in the elevated CAVI group.

	HR	95% CI	*p* Value	HR	95% CI	*p* Value
Hemoglobin (g/dL)	0.800	0.644–0.995	.045	0.802	0.653–0.984	.035
Coefficient of variation of systolic BP (mmHg)	1.180	0.990–1.400	.232			
Left ventricular end‐systolic dimension (mm)	0.976	0.937–1.020	.065			
Body mass index (kg/m^2^)				0.877	0.770–0.999	.484
Creatinine (mg/dL)				2.340	0.974–5.640	.574

*Note*: The multivariate analysis was performed applying Cox proportional hazards models.

Abbreviations: BP, blood pressure; CAVI, cardio ankle vascular index; CI, confidence interval; HR, hazard ratio; MACE, major adverse cardiovascular events.

**Table 2:** Multivariate analysis for prediction of MACE in the preserved CAVI group.

	HR	95% CI	*p* Value	HR	95% CI	*p* Value	HR	95% CI	*p* Value
Hemoglobin (g/dL)	0.783	0.615–0.988	.049	0.752	0.587–0.962	.023	0.754	0.589–0.964	.024
Diastolic BP (mmHg)	0.972	0.942–1.000	.072						
Cardiothoracic ratio (%)				1.090	0.966–1.220	.168			
Body mass index (kg/m^2^)							0.695	0.876–1.090	.695

*Note*: The multivariate analysis was performed applying Cox proportional hazards models.

Abbreviations: BP, blood pressure; CAVI, cardio ankle vascular index; CI, confidence interval; HR, hazard ratio; MACE, major adverse cardiovascular events.

### 3.4 Oral concomitant medications of both groups

Supporting Information: Table 5 shows the concomitant medications in the elevated and preserved CAVI groups. No significant difference in the rate of cardioprotective medication use was observed between patients with and without MACEs: classical HF medications (this study was conducted before the publication of the DAPA‐HF trial,^25^ and thus, sodium‐glucose cotransporter 2 inhibitors were not indicated at that time to be assessed). In the elevated CAVI group, β‐blockers were introduced at a rate of approximately 90%. The proportion of patients who received ≥50% of each maximum dose was low in both the MACE and non‐MACE groups; however, no significant difference was observed between patients without MACEs (18 of 61 patients [29.5%]) and those with MACEs (nine of 38 patients [23.7%]). Similarly, no significant difference in the use of RAS‐Is was observed between patients without MACEs (33 of 56 patients [58.9%]) and those with MACEs (18 of 31 patients [58.1%]). This tendency was similar in the preserved CAVI group, and no significant difference was observed (β‐blocker: non‐MACE: 29 of 80 patients [36.3%] vs. MACE: four of 13 patients [30.8%]; RAS‐I: non‐MACE: 40 of 62 patients [64.5%] vs. MACE: 7 of 11 patients [63.6%]).

## 4 DISCUSSION

### 4.1 Baseline characteristics in HF

HF severity was assessed using BNP and NHYA examinations. No significant differences were observed between patients with and without MACEs in the elevated and preserved CAVI groups. However, the subjects of this study had higher proportions of patients with NHYA III and higher BNP values than those of previous studies, indicating that patients with more severe HF were included in this study.^26^ Additionally, no significant differences in CS classification and HFpEF proportions were observed, and the type of HF was similar between the MACE and non‐MACE groups. Regarding the CS classification, approximately half of the study subjects were CS1, less than half of the study subjects were CS2, and the rest were CS3. HFpEF accounted for less than half of the study subjects. These findings were similar to those of previous studies.^26^, ^27^

### 4.2 BP and MACEs

In this study, no difference in BP was observed between patients with and without MACEs in the elevated CAVI group; however, the diastolic and mean BPs of patients with MACEs were significantly lower in patients with MACEs than in those without MACEs in the preserved CAVI group. Additionally, the CAVI of patients with MACEs was slightly elevated compared with that of patients without MACE, which may reflect arteriosclerosis; however, the difference did not reach statistical significance. The in‐hospital BPV of patients with MACEs was significantly higher than that of patients without MACEs in the elevated CAVI group. BPV has been reported to increase the risk of not only cardiovascular complications, including HF, in hypertension^28^ but also diabetes mellitus.^29^ BPV has short‐term (variability during all day), medium‐term (daily variability), and long‐term (visit‐to‐visit) values, and these reports evaluated visit‐to‐visit BPV. It has recently been reported that visit‐to‐visit BPV is also associated with the prognosis of HF.^30^, ^31^ In HFrEF, it has been reported that the prognosis worsens, regardless of the size of the variability; however, an increase in the variability range causes a poor prognosis.^9^ In‐hospital BPV is an indicator of short‐term BPV. This indicator is associated with the prognosis of arteriosclerotic diseases, such as coronary and peripheral artery diseases.^14^, ^15^ This result was also the case after coronary artery bypass grafting for coronary artery disease.^32^ In‐hospital BPV in HF was not investigated in previous studies; however, in this study, in‐hospital BPV was associated with the prognosis of HF in the elevated CAVI group. Patients with HF who have advanced arteriosclerosis may benefit from the evaluation of in‐hospital BPV.

### 4.3 Factors associated with the occurrence of MACEs

In both groups, BMI was significantly lower in patients with MACEs. The OBESICA study conducted in Japan showed that low BMI is associated with poor prognosis in HF.^33^ Furthermore, in Japan, the JCARE‐CRAD registry has reported similar results.^34^ Therefore, this result was similar to those of previous studies. However, in the elevated and preserved CAVI groups, no significant difference in the eGFR was observed between patients with and without MACEs, and the effect of renal function on anemia was limited. Usually, the left ventricle dilates as HF progresses. Generally, the occurrence of MACEs increases with the progression of HF^3^; however, the results of this study are contradictory. The number of subjects was considered the most important factor in this result, and the left ventricle cavity was excluded in the multivariate analysis. The use of therapeutic medications, the so‐called “cardioprotective medications,” which have been shown to improve the prognosis of HF, was evaluated; however, no significant difference was observed between the groups. Compared with previous studies, the introduction rate for each medication was higher in all groups. Moreover, the use of antianemic medications was so small that it could not be evaluated in this study.

### 4.4 Impact of anemia on HF

Several patients with HF have anemia. Anemia has been reported in one‐third to more than half of the Asian patients with HF in the Asian registry (ASIAN‐HF trial) in which our hospital also participated.^35^ Anemia was reported to be associated with a worsening HF prognosis,^7^ and similar results were obtained in this study. Anemia is often experienced due to decreased renal function caused by HF, also referred to as cardiorenal anemia syndrome. Because this anemia is accompanied by a decrease in erythropoietin production, erythropoiesis‐stimulating agents (ESAs) can be expected to improve Hb levels.^36^ However, although ESAs improved Hb levels, they did not improve HF prognosis.^37^ Furthermore, this result was similar even after using a high dose of ESAs, which sufficiently improved anemia.^38^ Moreover, HF is often associated with iron deficiency (ID) anemia. Many studies have evaluated the effectiveness of iron administration in improving HF. Improvement in ID anemia with intravenous iron supplements has been reported to be associated with improved motor performance and quality of life in patients with HFrEF^39^, ^40^; however, it was not associated with improved prognosis.^41^, ^42^ Anemia may be an important surrogate marker for evaluating HF prognosis. However, as described above, neither ESAs nor iron supplementation improves the prognosis of HF; therefore, addressing anemia as a therapeutic target in HF may be difficult. Furthermore, it has been reported that hypoxia‐inducible factor prolyl hydroxylase, a new medication for treating anemia, improved BNP in patients with HF,^43^ and these therapeutic medications may provide new perspectives on treating anemia in patients with HF. Additionally, in this study, anemia was a factor more related to HF prognosis than BPV. Anemia combined with low BP has been reported to predict the early necessity of ventricular assist device implantation.^44^ In contrast, no studies have investigated whether BP or BPV and anemia are associated with HF prognosis until this study.

### 4.5 Study limitations

The most important study limitation is the number of study subjects. This study was a retrospective, observational, single‐center study. The sample size was small because vascular function was not evaluated in all patients. Besides the CAVI, reactive hyperemia, flow‐mediated vasodilation, peripheral arterial tonometry, and PWV are known vascular function evaluation methods.^45^ However, in this study, only the CAVI was evaluated because the number of subjects for whom examinations other than the CAVI were performed was even smaller. Although the CAVI has advantages, such as being simpler and less influenced by BP than other methods, one of the limitations of this study was that only the CAVI was evaluated. If many subjects were included in this study, increasing the number of factors included in the multivariate analysis is possible even after grouping into the preserved and elevated CAVI groups, and it was possible that factors other than anemia also showed statistically significant differences.

Additionally, in this study, no patients had autonomic disorder; however, the possibility that underlying diseases and medications affected BPV cannot be denied. Furthermore, the data for this study were extracted from medical charts. Therefore, we could not evaluate frailty associated with HF prognosis because no description of the grip strength or quality of life table/score was provided. Because left ventricular diastolic dysfunction was not evaluated in all patients, and this study was retrospective, the data that could be extracted were limited.

### 4.6 Future directions

To address the aforementioned limitations conducting studies with a large number of subjects is essential. The concomitant use of other arteriosclerosis assessment methods is also useful. We also evaluated short‐term BPV during hospitalization (in‐hospital BPV); however, BPV evaluation after discharge in these subjects may also be useful. Further clinical prospective studies with a larger number of consecutive subjects are required to confirm the results of this study.

## 5 CONCLUSION

Anemia was independently associated with the prognosis of HF with or without arteriosclerosis.

## AUTHOR CONTRIBUTIONS

Shunsuke Kiuchi contributed to the study concept and the acquisition of the data. All authors reviewed the raw data and contributed to the analysis and interpretation. Shunsuke Kiuchi drafted the manuscript and Shinji Hisatake, Takayuki Kabuki, Shintaro Dobashi, Yoshiki Murakami, and Takanori Ikeda revised it. Finally, all authors approved the final manuscript.

## CONFLICTS OF INTEREST STATEMENT

T. I. received research funds and lecture fees from Daiichi‐Sankyo, Co., Ltd., Bayer Healthcare, Co., Ltd., and Bristol‐Myers Squibb, Co., Ltd. The remaining author declares no conflict of interest.

## Supporting information

Supporting information.

## Data Availability

The data sets used and/or analyzed during the current study are available from the corresponding author on reasonable request.
