# Supplementary material for: Importance of anemia in heart failure over blood pressure variability
Source: Clin Cardiol. 2023 Aug 30;46(12):1495–503. doi: 10.1002/clc.24141 (PMC10716338; doi:10.1002/clc.24141)
Supplement: Supplementary file 1 — Supporting information. [file CLC-46-1495-s001.doc]

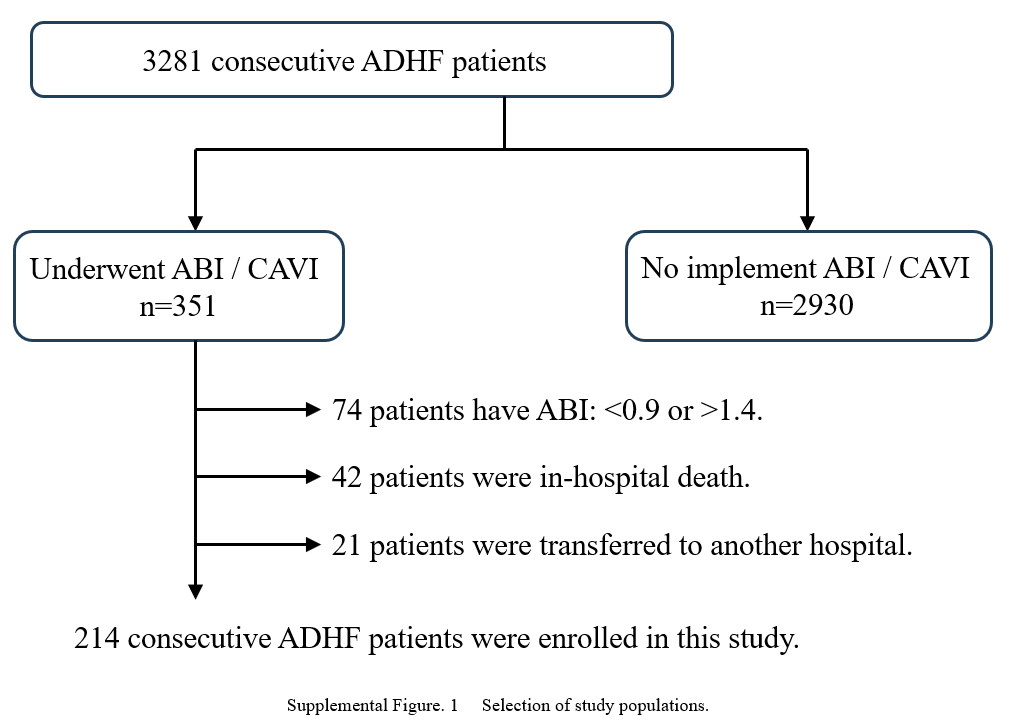


Supplemental Table 1. Patient’s clinical and physical profiles in the elevated CAVI group

|  | Non-MACE  (n = 70) | MACE  (n = 43) | *P* value |
| --- | --- | --- | --- |
| Age (years) | 70.6 ± 9.8 | 71.7 ± 9.9 | 0.554 |
| Male (n, %) | 46, 65.7 | 25, 58.1 | 0.365 |
| Height (cm) | 161.5 ± 9.2 | 159.7 ± 10.5 | 0.335 |
| Weight (kg) | 59.8 ± 15.0 | 53.7 ± 10.8 | 0.021 |
| Body mass index (kg/m^2^) | 22.8 ± 4.3 | 20.9 ± 2.7 | 0.012 |
| NYHA class (II/ III/ IV) | 1 / 50 / 19 | 0 / 34 / 9 | 0.584 |
| Hospital stay (days) | 20.7 ± 13.9 | 21.4 ± 17.4 | 0.813 |
| CS class (I / II / III) | 39 / 30 / 1 | 23 / 17 / 3 | 0.483 |
| Systolic BP (mmHg) | 149.1 ± 29.5 | 149.6 ± 39.0 | 0.939 |
| Diastolic BP (mmHg) | 88.2 ± 23.4 | 85.7 ± 22.4 | 0.576 |
| Pulse pressure (mmHg) | 60.9 ± 21.1 | 63.9 ± 23.5 | 0.484 |
| Mean BP (mmHg) | 108.5 ± 23.6 | 107.0 ± 26.8 | 0.755 |
| Standard deviation of systolic BP (mmHg) | 7.88 ± 2.78 | 9.16 ± 4.21 | 0.054 |
| Coefficient of variation of systolic BP (mmHg) | 6.97 ± 1.98 | 8.04 ± 3.08 | 0.027 |
| Heart rate (bpm) | 101.5 ± 29.3 | 93.1 ± 25.2 | 0.125 |
| Medical history of hypertension (n %) | 43, 61.4 | 32, 74.4 | 0.158 |
| Medical history of diabetes (n %) | 22, 31.4 | 15, 34.9 | 0.707 |
| Medical history of AF/Paf (n %) | 22, 31.4 | 13, 30.2 | 0.918 |
| Medical history of CKD (n %) | 43, 61.4 | 37, 83.7 | 0.005 |
| Medical history of hemodialysis (n %) | 0, 0 | 0, 0 |  |

CAVI: cardio ankle vascular index, MACE: major adverse cardiovascular events, NYHA: New York Heart Association, CS: clinical scenario, BP: blood pressure, AF/Paf: atrial fibrillation/Paroxysmal atrial fibrillation, CKD: chronic kidney disease. Continuous data are expressed as the mean ± standard deviation or error. P-values were determined using the unpaired t-test.

Supplemental Table 2. Patient’s clinical and physical profiles in the preserved CAVI group

|  |  | Non-MACE  (n = 87) | MACE  (n = 14) | *P* value |
| --- | --- | --- | --- | --- |
|  | Age (years) | 60.5 | 72.5 | 0.083 |
|  | Male (n, %) | 69, 79.3 | 7, 50.0 | 0.079 |
|  | Height (cm) | 167.7 | 154.0 | 0.014 |
|  | Weight (kg) | 66.3 | 51.7 | 0.008 |
|  | Body mass index (kg/m^2^) | 24.1 | 20.8 | 0.038 |
|  | NYHA class (II/ III/ IV) | 1 / 65 / 21 | 0 / 11 / 3 | 0.665 |
|  | Hospital stay (days) | 15 | 18 | 0.482 |
|  | CS class (I / II / III) | 44 / 37 / 6 | 5 / 8 / 1 | 0.403 |
|  | Systolic BP (mmHg) | 141.0 | 136.5 | 0.147 |
|  | Diastolic BP (mmHg) | 90.0 | 70.5 | 0.021 |
|  | Pulse pressure (mmHg) | 54.0 | 61.0 | 0.727 |
|  | Mean BP (mmHg) | 105.7 | 92.0 | 0.032 |
|  | Standard deviation of systolic BP (mmHg) | 7.85 | 7.21 | 0.072 |
|  | Coefficient of variation of systolic BP (mmHg) | 7.28 | 6.28 | 0.107 |
|  | Heart rate (bpm) | 98.0 | 103.0 | 0.423 |
| Medical history of hypertension (n %) | | 54, 62.0 | 11. 78.6 | 0.323 |
| Medical history of diabetes (n %) | | 22, 25.3 | 2, 14.3 | 0.510 |
| Medical history of AF/Paf (n %) | | 10, 11.5 | 2. 14.3 | 0.867 |
| Medical history of CKD (n %) | | 37, 42.5 | 10. 71.4 | 0.084 |
| Medical history of hemodialysis (n %) | | 3, 3.4 | 0, 0 | 0.836 |

CAVI: cardio ankle vascular index, MACE: major adverse cardiovascular events, NYHA: New York Heart Association, CS: clinical scenario, BP: blood pressure, AF/Paf: atrial fibrillation/Paroxysmal atrial fibrillation, CKD: chronic kidney disease. Continuous data are expressed as the median. P-values were determined using the Mann–Whitney U test.

Supplemental Table 3. Examinations in the elevated CAVI group

|  | Non-MACE  (n = 70) | MACE  (n = 43) | *P* value |
| --- | --- | --- | --- |
| Left atrial dimension (mm) | 43.0 ± 8.6 | 42.3 ± 8.0 | 0.702 |
| Left ventricular end-diastolic dimension (mm) | 58.0 ± 9.2 | 51.9 ± 8.4 | 0.001 |
| Left ventricular end-systolic dimension (mm) | 44.9 ± 11.1 | 39.3 ± 11.0 | 0.010 |
| Ejection fraction (%) | 44.9 ± 15.9 | 48.2 ± 18.9 | 0.314 |
| The proportion of HFpEF (n, %) | 23. 32.9 | 22, 51.2 | 0.054 |
| Ankle brachial index (right) | 1.12 ± 0.10 | 1.09 ± 0.09 | 0.120 |
| Ankle brachial index (left) | 1.11 ± 0.10 | 1.09 ± 0.10 | 0.266 |
| Cardio ankle vascular index | 10.19 ± 1.11 | 10.06 ± 0.83 | 0.511 |
| Sodium (mg/dl) | 139.4 ± 3.8 | 139.4 ± 3.5 | 0.947 |
| Potassium (mg/dl) | 4.1 ± 0.6 | 4.1 ± 0.5 | 0.788 |
| AST (IU/L) | 49.3 ± 51.3 | 48.7 ± 44.3 | 0.948 |
| ALT (IU/L) | 41.7 ± 44.8 | 38.7 ± 50.0 | 0.750 |
| LDH (IU/L) | 305.4 ± 80.7 | 332.7 ± 113.3 | 0.137 |
| BUN (mg/dl) | 19.6 ± 7.1 | 25.2 ± 10.3 | 0.001 |
| Creatinine (mg/dL) | 1.04 ± 0.38 | 1.24 ± 0.61 | 0.029 |
| eGFR (ml/min/1.73 m^2^) | 56.6 ± 17.9 | 49.2 ± 23.3 | 0.061 |
| LDL cholesterol (mg/dl) | 101.6 ± 4.8 | 101.3 ± 4.0 | 0.963 |
| HDL cholesterol (mg/dl) | 52.3 ± 2.4 | 53.2 ± 2.3 | 0.779 |
| Triglyceride (mg/dl) | 94.3 ± 13.5 | 94.8 ± 8.3 | 0.972 |
| Fasting glucose (mg/dl) | 154.5 ± 11.7 | 181.7 ± 11.7 | 0.121 |
| Hemoglobin A1C (%) | 6.6 ± 0.2 | 6.6 ± 0.2 | 0.994 |
| Hemoglobin (g/dL) | 13.6 ± 1.8 | 12.4 ± 2.4 | 0.003 |
| The proportion of anemia (n, %) | 20. 28.6 | 22, 51.2 | 0.016 |
| Platelet (×10^3^/µL) | 190.9 ± 85.0 | 207.5 ± 65.0 | 0.245 |
| Brain natriuretic peptide (pg/mL) | 942.5 ± 608.6 | 1050.8 ± 707.7 | 0.390 |
| Cardiothoracic ratio (%) | 60.5 ± 5.7 | 62.6 ± 5.7 | 0.057 |

CAVI: cardio ankle vascular index, MACE: major adverse cardiovascular events, HFpEF: heart failure with preserved ejection fraction, AST: aspartate aminotransferase, ALT: alanine aminotransferase, LDH: lactate dehydrogenase, BUN: blood urea nitrogen, eGFR: estimated glomerular filtration rate, LDL: low density lipoprotein, HDL: high density lipoprotein. Continuous data are expressed as the mean ± standard deviation or error. P-values were determined using the unpaired t-test.

Supplemental Table 4. Examinations in the preserved CAVI group

|  | Non-MACE  (n = 87) | MACE  (n = 14) | *P* value |
| --- | --- | --- | --- |
| Left atrial dimension (mm) | 43.5 | 40.3 | 0.236 |
| Left ventricular end-diastolic dimension (mm) | 61.1 | 57.4 | 0.314 |
| Left ventricular end-systolic dimension (mm) | 48.3 | 42.9 | 0.205 |
| Ejection fraction (%) | 40.6 | 54.3 | 0.616 |
| The proportion of HFpEF (n, %) | 29, 33.3 | 7, 50 | 0.318 |
| Ankle brachial index (right) | 1.11 | 1.13 | 0.495 |
| Ankle brachial index (left) | 1.11 | 1.10 | 0.829 |
| Cardio ankle vascular index | 7.55 | 7.58 | 0.536 |
| Sodium (mg/dl) | 139.0 | 139.5 | 0.178 |
| Potassium (mg/dl) | 4.0 | 4.1 | 0.455 |
| AST (IU/L) | 33.0 | 28.0 | 0.526 |
| ALT (IU/L) | 31.0 | 19.0 | 0.049 |
| LDH (IU/L) | 296.5 | 292.0 | 0.827 |
| BUN (mg/dl) | 18.0 | 16.5 | 0.536 |
| Creatinine (mg/dL) | 0.97 | 0.94 | 0.840 |
| eGFR (ml/min/1.73 m^2^) | 62.9 | 55.9 | 0.275 |
| LDL cholesterol (mg/dl) | 96.0 | 101.5 | 0.815 |
| HDL cholesterol (mg/dl) | 51.5 | 49.1 | 0.599 |
| Triglyceride (mg/dl) | 84.0 | 81.5 | 0.409 |
| Fasting glucose (mg/dl) | 127.0 | 131.0 | 0.696 |
| Hemoglobin A1C (%) | 5.7 | 5.9 | 0.715 |
| Hemoglobin (g/dL) | 14.0 | 12.4 | 0.019 |
| The proportion of anemia (n, %) | 20, 22.9 | 8, 57.1 | 0.041 |
| Platelet (×10^3^/µL) | 208.5 | 221.0 | 0.440 |
| Brain natriuretic peptide (pg/mL) | 767.6 | 848.3 | 0.623 |
| Cardiothoracic ratio (%) | 60.7 | 63.1 | 0.036 |

CAVI: cardio ankle vascular index, MACE: major adverse cardiovascular events, HFpEF: heart failure with preserved ejection fraction, AST: aspartate aminotransferase, ALT: alanine aminotransferase, LDH: lactate dehydrogenase, BUN: blood urea nitrogen, eGFR: estimated glomerular filtration rate, LDL: low density lipoprotein, HDL: high density lipoprotein. Continuous data are expressed as the median. P-values were determined using the Mann–Whitney U test.

Supplemental Table 5a. Concomitant medications in the elevated CAVI group

|  | Non-MACE  (n = 70) | MACE  (n = 43) | *P* value |
| --- | --- | --- | --- |
| Administration rate of β-blockers at discharge (n, %) | 61, 87.1 | 38, 88.4 | 0.849 |
| Administration rate of RAS-Is at discharge (n, %) | 56, 80.0 | 31, 72.1 | 0.337 |
| Administration rate of MRAs at discharge (n, %) | 42, 60.0 | 23, 53.5 | 0.501 |

CAVI: cardio ankle vascular index, MACE: major adverse cardiovascular events, RAS-I: renin–angiotensin–aldosterone system inhibitor, MRA: mineral corticoid receptor antagonist. P-values were determined using the unpaired t-test.

Supplemental Table 5b. Concomitant medications in the preserved CAVI group

|  | Non-MACE  (n = 87) | MACE  (n = 14) | *P* value |
| --- | --- | --- | --- |
| Administration rate of β-blockers at discharge (n, %) | 80, 92.0 | 13, 92.9 | 0.957 |
| Administration rate of RAS-Is at discharge (n, %) | 62, 71.3 | 11, 78.6 | 0.662 |
| Administration rate of MRAs at discharge (n, %) | 60, 69.0 | 8, 57.1 | 0.479 |

CAVI: cardio ankle vascular index, MACE: major adverse cardiovascular events, RAS-I: renin–angiotensin–aldosterone system inhibitor, MRA: mineral corticoid receptor antagonist. P-values were determined using the Mann–Whitney U test.
